# Characteristics of Cardiac Rehabilitation for Older Patients in a Japanese Rehabilitation Hospital

**DOI:** 10.7759/cureus.80939

**Published:** 2025-03-21

**Authors:** Tomohiro Matsuo, Takuro Ohtsubo, Tomoki Yanase, Katsuhiro Ueno, Shuichi Kozawa, Yosuke Morimoto

**Affiliations:** 1 Department of Rehabilitation, Nishi Memorial Port Island Rehabilitation Hospital, Kobe, JPN; 2 Department of Cardiology, Nishi Memorial Port Island Rehabilitation Hospital, Kobe, JPN; 3 Department of Physical Therapy, Faculty of Rehabilitation, Kobe Gakuin University, Kobe, JPN

**Keywords:** cardiac rehabilitation, cardiac rehabilitation components, older patients, post-acute care, rehabilitation hospital

## Abstract

Background

Japan’s aging population faces a rising prevalence of cardiovascular disease (CVD), resulting in an increased demand for specialized cardiac rehabilitation (CR). This study investigated the characteristics of older patients and the current status of CR in Japanese convalescent rehabilitation hospitals.

Materials and methods

This retrospective cohort study enrolled 107 patients who were transferred to a rehabilitation hospital following acute CVD treatment. The patients were divided into an older group (aged ≥80 years) and a control group (aged <80 years). Clinical characteristics, physical and cognitive function, exercise tolerance, activities of daily living (ADL), health-related quality of life (HR-QoL), and CR components were collected and analyzed.

Results

Despite shorter aerobic exercise duration (median, 9.8 (IQR, 0.4-19.2) vs. 20.6 (11.7-29.8) min, *P* < 0.001), the older group demonstrated significant improvements in physical function, ADL, and HR-QoL by discharge. Both groups showed gains in functional independence measures, physical performance, strength, gait speed, and endurance. While the older group started with lower baseline physical performance and required a higher level of care at discharge, their improvements in physical function, exercise tolerance, ADL, and HR-QoL were comparable to those in the control group.

Conclusion

CR programs for older patients in rehabilitation hospitals, although characterized by relatively short durations of aerobic exercise, may contribute to improved functional outcomes. These results highlight the importance of developing and implementing CR programs for older patients and support their potential for broader application in addressing the needs of Japan’s aging population.

## Introduction

Japan’s rapidly aging society, with 29.1% of the population aged ≥65 years, including 10.1% aged ≥80 years (octogenarians) in 2023 [1], is a key contributor to the rising prevalence of cardiovascular diseases (CVD) [2]. As the population ages and CVD treatment advances, many patients presenting to acute care hospitals experience significant deconditioning, multimorbidity, and impaired performance of activities of daily living (ADL) [3]. These patients frequently exhibit complex conditions, such as frailty, sarcopenia, and multimorbidity, often resulting in discharge from acute care hospitals before achieving sufficient recovery of ADL [4].

Octogenarians with heart failure were observed to have reduced ADL at both admission and discharge, higher dependence on long-term care insurance, and prolonged hospital stays [5]. Furthermore, a large Japanese registry of physical therapy in older patients (median age, 83 years) with heart failure (J-Proof HF Registry) reported that 37.1% of patients admitted to acute care hospitals for heart failure had hospitalization-associated disability (HAD) [6]. Consequently, these older patients are more likely to require transfer to convalescent rehabilitation hospitals to continue cardiac rehabilitation (CR). A convalescent rehabilitation hospital provides intensive rehabilitation for patients who require ongoing medical, social, and psychological support after the acute phase of illness or injury. The primary goal is to facilitate physical and cognitive recovery, enabling patients to regain independence and reintegrate into their homes and communities.

Continuous CR in a convalescent rehabilitation hospital has become increasingly important, particularly in light of the FY 2022 Revision of Medical Fees, which added requirements for calculating convalescent rehabilitation coverage. Although CR was reported effective in rehabilitation hospitals [7], most participants were older patients, necessitating the development of individualized treatment plans tailored to the specific characteristics of older people [8].

In clinical practice, many older patients transferred to rehabilitation hospitals encounter challenges in implementing the guideline-recommended CR programs [4], particularly aerobic exercise. This indicates that CR for older patients needs to be highly individualized, especially for those who struggle with aerobic exercise. A subanalysis of the J-Proof HF Registry reported low engagement in aerobic exercise; conversely, walking and muscle strength training were more commonly employed [9]. However, the actual CR and subsequent outcomes for individual older patients, especially octogenarians, in convalescent rehabilitation hospitals are vague.

Thus, the aim of this study was to investigate the characteristics, CR content, physical function, and ADL outcomes at discharge in octogenarians with CVD undergoing rehabilitation in a convalescent rehabilitation hospital. By examining the impact of individualized CR on functional outcomes in this population, the findings may contribute critical data to inform CR for Japan's rapidly aging society, with implications for clinical practice.

## Materials and methods

Study design and population

This retrospective cohort study was conducted between December 2020 and March 2024, enrolling consecutive patients who underwent conservative management or surgical intervention for CVD at an acute care facility. Following initial treatment, these individuals were transferred to Nishi Memorial Port Island Rehabilitation Hospital for comprehensive CR. The study included patients aged 18 years or older. The actual age range of the participants in our sample was 45 to 96. However, individuals with significant ambulatory limitations due to conditions such as severe consciousness disturbances, hemiplegia secondary to cerebrovascular events, spinal cord infarction resulting in paraplegia, critical limb ischemia, or prior lower extremity amputation were excluded. Further exclusion criteria encompassed patients who succumbed during hospitalization, were unable to continue inpatient rehabilitation, required re-transfer to an acute care facility, or declined participation. Patients were divided into two groups based on age: those aged <80 years (control group) and those aged ≥80 years (older group).

Ethical approval for this study was obtained from the Nishi Memorial Port Island Rehabilitation Hospital Ethics Committee (Approval No. 16). Participants were fully informed about the purpose of data usage and procedures for opting out. Those who opted out had their decisions respected, and data collection was discontinued. This study adhered to the ethical principles outlined in the Declaration of Helsinki, ensuring the protection and rights of human subjects throughout the research.

Data collection prior to hospitalization and upon transfer to the rehabilitation hospital

Information regarding pre-admission status, including caregiver presence, ADL as measured by the Barthel Index (BI), and frailty assessment via the Kihon Checklist (KCL) [10], was recorded. Upon transfer to the rehabilitation hospital, the following demographic and clinical parameters were documented: age, sex, body mass index (BMI), primary indication for CR (heart failure, ischemic heart disease, including angina pectoris and acute coronary syndrome, cardiovascular surgery, such as coronary artery bypass grafting and valvular procedures, aortic surgery-encompassing vascular replacement and stent grafting, or combined cardiovascular and aortic procedures), New York Heart Association functional classification (NYHA class), Charlson comorbidity index, echocardiographic assessments (left ventricular ejection fraction, left atrial dimension, and ratio of early diastolic transmitral flow velocity to early diastolic mitral annular tissue velocity), laboratory findings (serum hemoglobin, albumin, C-reactive protein, B-type natriuretic peptide, and estimated glomerular filtration rate), and length of stay in the acute care hospital.

Assessment during inpatient rehabilitation

A team comprising a physical therapist and an occupational therapist evaluated ADL levels at both hospital transfer and discharge. Functional independence was determined using the functional independence measure (FIM) [11], an 18-item tool assessing both motor (13 items) and cognitive (five items) domains on a seven-point scale, where lower scores indicate greater dependence.

Physical function assessments were conducted at admission and discharge. Handgrip strength was measured using a digital handgrip meter (T-2177, TOEI LIGHT Co., Ltd., Tokyo, US), with the highest of two trials recorded. Knee extension strength was determined using a handheld dynamometer (μTas F-1, Anima Co., Ltd., New York, US), with isometric force recorded at 90-degree knee flexion while seated. The greater of two attempts was divided by body weight to yield a percentage value (%BW). Gait speed was assessed over a 10-meter walk, and performance in balance, short-distance walking, and sit-to-stand transitions was evaluated using the short physical performance battery (SPPB) [12].

Exercise tolerance was assessed at both time points using the six-minute walk test (6MWT), conducted along a 20-meter, obstacle-free corridor. The maximum distance covered within six minutes was recorded in accordance with American Thoracic Society guidelines [13]. Cognitive function was examined using the Japanese version of the Mini-Mental State Examination (MMSE) [14], with scores ranging from 0 to 30, where higher scores denote better function. Health-related quality of life (HR-QoL) was quantified using the Japanese version of the EuroQol five-dimension five-level (EQ-5D-5L) instrument [15]. Nutritional status was assessed using the Mini Nutritional Assessment-Short Form (MNA-SF) [16].

The length of hospital stays, patient outcomes at the rehabilitation hospital, level of care at discharge, and details of CR components and their implementation times were extracted from the medical records. The level of care was assessed using an eight-level certification system, which includes support levels one and two and care levels 1-5 [17]. Independence was defined as level 0, whereas levels 1-7 indicated varying degrees of care required.

CR program

The CR program adhered to the guidelines established by the Japanese Circulation Society [4]. Although cardiopulmonary exercise testing based on anaerobic thresholds is recommended for determining exercise intensity [4], its feasibility in patients undergoing rehabilitation following acute hospitalization is limited due to functional impairments and comorbid conditions. Thus, exercise intensity was predominantly monitored using the Borg Perceived Exertion Scale [18]. Rehabilitation intensity and duration were adjusted according to the patient's clinical progress. Individualized rehabilitation sessions were scheduled daily, lasting up to three hours per day. All patients received physical and occupational therapy. Individualized rehabilitation programs were designed based on each patient's initial physical, cognitive, and ADL status at the time of transfer. These programs were structured to address functional recovery needs and anticipated post discharge roles, and were regularly reassessed and modified to optimize rehabilitation outcomes. Physical therapy emphasized aerobic and resistance training, with additional gait and balance training as needed. Occupational therapy focused on ADL and instrumental ADL exercises, upper limb function, and cognitive activities. Speech therapy was provided upon a physician's recommendation. The rehabilitation program encompassed six key elements: aerobic exercise, resistance training, ADL training (including mobility and instrumental ADL exercises), balance training, conditioning training (such as stretching, breathing exercises, and accessory respiratory muscle conditioning), and supplementary interventions. Educational components addressed physical activity, nutrition, and stress management to support long-term adherence post-discharge. The average duration of each component was determined based on the length of rehabilitation hospital stay.

Statistical analysis

Clinicodemographic characteristics of the older and control groups were compared using either an unpaired t-test or Mann-Whitney U-test, depending on the data distribution, and a chi-squared test for categorical variables. Clinical outcomes, including ADL, physical function, exercise tolerance, cognitive function, HR-QoL, and nutritional status, were compared between pre and post CR using either the paired-sample t-test or the Wilcoxon signed-rank test, based on the data distribution. Two-way analysis of variance (ANOVA) was used to assess changes in clinical outcomes and the interaction effects of group differences (older versus control). When a statistically significant interaction was identified, the Bonferroni method was used for post hoc comparisons. No adjustment was made for multiplicity, as this was an exploratory analysis. Analyses were performed with IBM SPSS Statistics for Windows version 28.0 (IBM Corp., Armonk, NY, USA). A *P*-value of 0.05 was considered statistically significant.

## Results

Patient demographics

**Figure 1:**
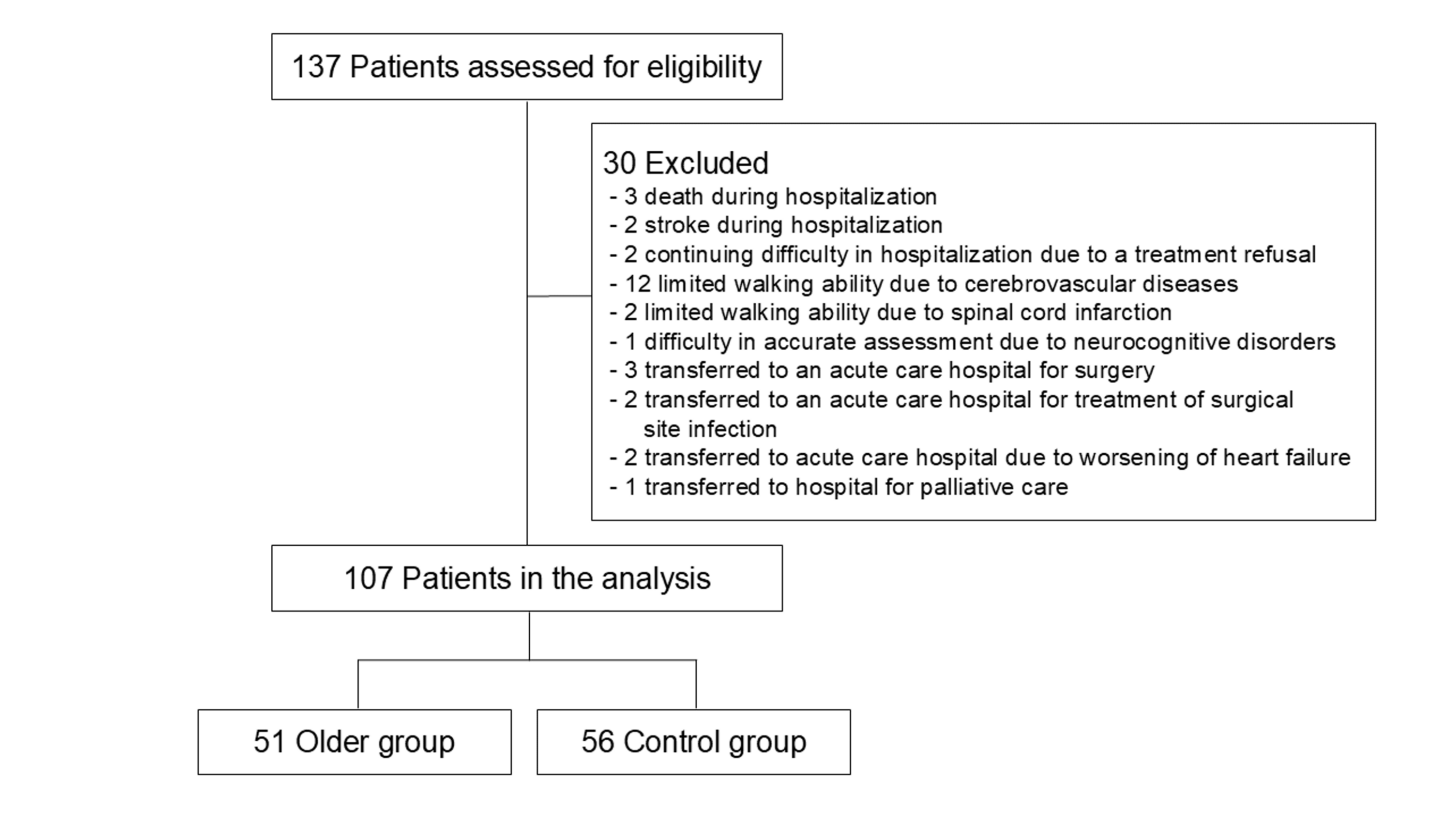
Flowchart depicting study enrollment and exclusion criteria

A total of 137 patients transferred for post-acute CVD rehabilitation were screened, with 107 ultimately included in the analysis following the application of selection criteria. Of these, 56 were assigned to the control group and 51 to the older group. The participant flowchart is illustrated in Figure 1, and baseline characteristics are summarized in Table 1.

**Table 1:** Baseline characteristics of the study patients *, *P* < 0.001, †, *P* < 0.01, ‡, *P* < 0.05 Results expression as median (IQR) or n (%) as indicated BI, Barthel index; KCL, Kihon checklist; LOS, length of hospital stays; CCI, Charlson comorbidity index; NYHA class, New York Heart Association functional classification; LVEF, left ventricular ejection fraction; LAD, left atrial dimension; E/e’, early diastolic transmitral flow velocity to early diastolic mitral annular tissue velocity ratio; eGFR, estimated glomerular filtration rate; CRP, C-reactive protein; BNP, brain natriuretic peptide; CR, cardiac rehabilitation; ADL, activities of daily living

	All ( n = 107 )	Older group ( n = 51 )	Control group ( n = 56 )
Basic data			
Age (years)	79 (73 – 85)	85 (82 – 88) *	73.0 (68.0 – 76.3)
Male, n (%)	44 (41.1)	15 (29.4) ^‡^	29 (51.8)
Body Mass Index (kg/m^2^)	20.6 (17.9 – 22.5)	19.7 (16.8 – 21.9)	21.3 (19.1 – 22.7)
BI before hospitalization	100 (100 – 100)	100 (96.3 – 100)	100 (100 – 100)
KCL before hospitalization	7 (4 – 13)	10 (5 – 14) ^†^	6 (4 – 9)
Living alone, n (%)	33 (30.8)	22 (43.1) ^†^	11 (19.6)
Principal disease, n (%)			
Heart failure	18 (16.8)	11 (21.6)	7 (12.5)
Ischemic heart disease	11 (10.3)	8 (15.7)	3 (5.4)
Cardiovascular surgery	35 (32.7)	16 (31.4)	19 (33.9)
Aortic surgery	28 (26.2)	11 (21.6)	17 (30.4)
Combined surgery (Cardiovascular and aortic surgery)	15 (14.0)	5 (9.8)	10 (17.9)
LOS before transfer, days	29.0 (22.0 – 41.5)	29.0 (22.0 – 36.5)	29.5 (22.8 – 44.3)
Rehabilitation hospital transfer data			
CCI	3.0 (2.0 – 4.0)	3.0 (2.0 – 4.5)	3.0 (2.0 – 4.0)
NYHA class, n (%)			
Class 1	0 (0)	0 (0)	0 (0)
Class 2	14 (13.1)	3 (5.9)	11 (19.6)
Class 3	86 (80.4)	44 (86.3)	42 (75.0)
Class 4	7 (6.5)	4 (7.8)	3 (5.4)
Echocardiographic findings			
LVEF, %	58.7 (45.4 – 62.0)	59.0 (48.0 – 62.1)	57.9 (36.8 – 61.9)
LAD, mm	37.0 (33.0 – 42.0)	37.5 (34.0 – 43.3)	37.0 (32.7 – 42.0)
E/e’	12.5 (9.3 – 16.6)	13.3 (9.8 – 17.9)	12.0 (9.0 – 16.0)
Laboratory data			
Hemoglobin, g/dL	11.1 (10.2 – 12.1)	11.3 (10.3 – 12.1)	11.0 (9.9 – 12.1)
Albumin, g/mL	3.3 (3.1 – 3.6)	3.3 (3.1 – 3.6)	3.3 (3.1 – 3.6)
CRP, mg/dL	0.76 (0.20 – 1.71)	0.43 (0.15 – 1.32) ^‡^	0.88 (0.30 – 2.26)
BNP, pg/mL	143.0 (80.7 – 269.7)	142.0 (85.3 – 249.2)	148.3 (77.5 – 276.3)
eGFR, mL/min/1.73m^2^	55.3 (41.9 – 67.6)	51.1 (38.9 – 65.6)	57.6 (43.9 – 72.0)
Medication			
Beta-blocker, n (%)	79 (73.8)	44 (86.3)	35 (62.5)
ACEi/ARB, n (%)	34 (31.8)	16 (31.4)	18 (32.1)
Diuretics, n (%)	57 (53.3)	28 (54.9)	29 (51.8)
Rehabilitation hospital process			
Total CR time, min/day	137.8 (126.9 – 148.4)	139.7 (122.7 – 148.7)	136.6 (128.1 – 147.7)
Aerobic exercise time, min/day	16.9 (6.9 – 26.3)	9.8 (0.4 – 19.2) *	20.6 (11.7 – 29.8)
Resistance training time, min/day	25.0 (19.4 – 30.7)	26.5 (20.1 – 34.4)	23.2 (18.6 – 29.1)
ADL training time, min/day	20.7 (14.4 – 29.3)	22.2 (16.5 – 30.8)	18.5 (10.7 – 26.4)
Balance training time, min/day	3.0 (0.6 – 7.7)	3.2 (0.6 – 5.8)	2.7 (0.5 – 8.2)
Conditioning training time, min/day	31.1 (20.3 – 40.3)	31.7 (21.3 – 39.6)	28.0 (19.9 – 40.9)
LOS, days	71.0 (44.0 – 88.0)	77.0 (57.0 – 87.5)	60.5 (32.8 – 88.0)
Temporary transfer to an acute care hospital, n (%)	9 (8.4)	4 (7.8)	5 (8.9)
Care level at discharge	2 (1 – 5)	4 (2 – 5) ^†^	1 (0 – 4)
Home discharge, n (%)	96 (89.7)	43 (84.3)	53 (94.6)

Comparison of older and control groups

The older group includes a higher proportion of female patients (70.6% vs. 48.2%, *P* = 0.019), higher preadmission KCL scores (median, 10 vs. 6, *P* = 0.002), and a greater percentage of individuals living alone (43.1% vs. 19.6%, *P* = 0.009). At discharge, this group required a higher level of care at discharge (4 vs. 1, *P* = 0.003). In addition, the C-reactive protein (CRP) levels were significantly lower in the older group (*P* = 0.043) (Table 1). Functional measures at transfer showed that the older group had slower gait speed (0.68 vs. 0.87 m/s, *P* < 0.001), lower hand grip strength (12.7 vs. 16.2 kg, *P* < 0.001), and shorter 6-min walking distance (6MD) (120.0 vs. 228.5 m, *P* = 0.002) than the control group. By discharge, the control group demonstrated a faster gait speed (1.19 vs. 0.92 m/s, *P* < 0.001), stronger hand grip strength (18.6 vs. 13.9 kg, *P* < 0.001), and longer 6MD (363.0 vs. 250.0 m,* P* < 0.001).

Rehabilitation dose

**Figure 2:**
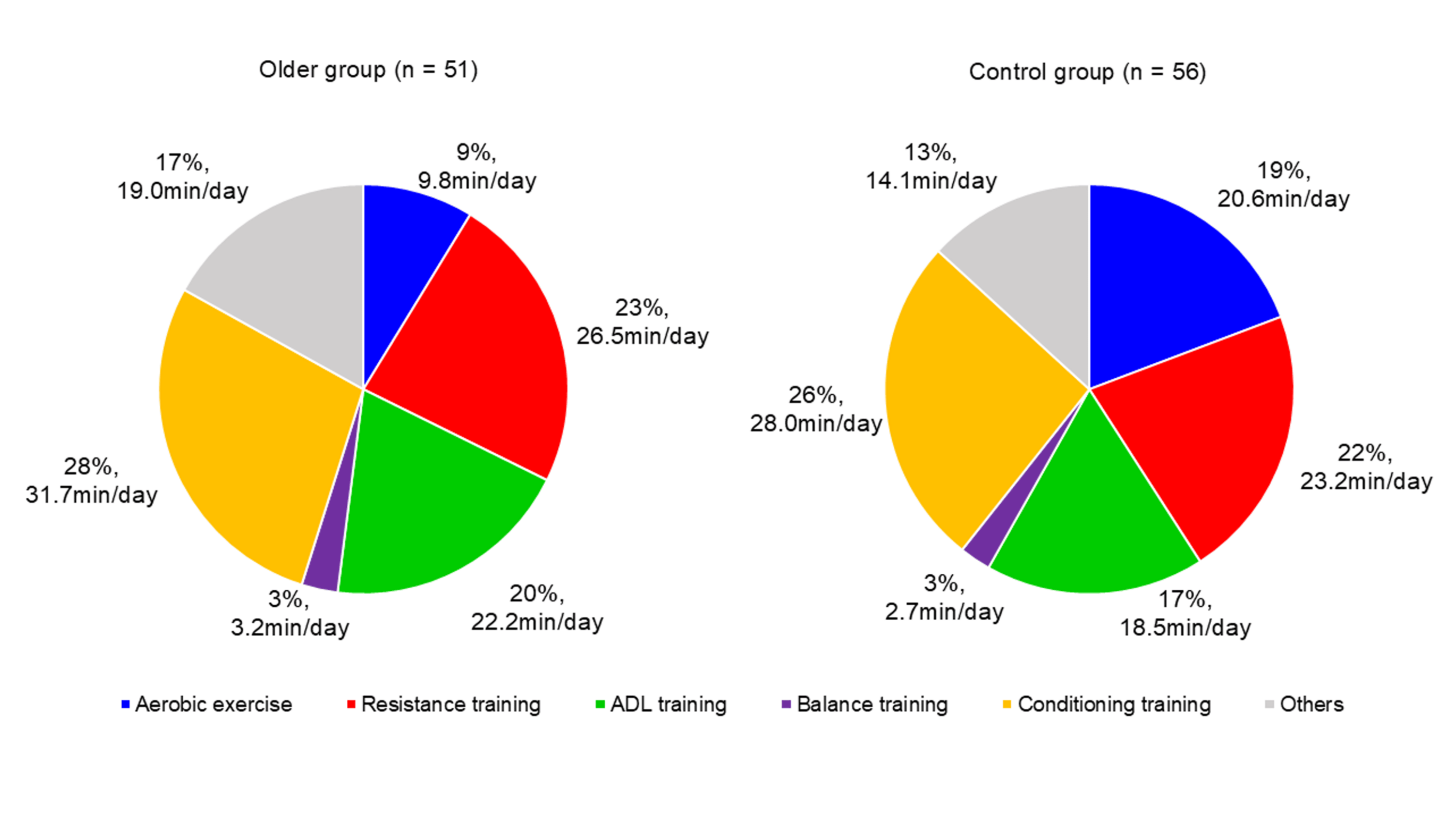
Content and proportion of cardiac rehabilitation programs in a rehabilitation hospital Percentages represent the proportion of total daily rehabilitation time allocated to each type of exercise. The median exercise duration (in minutes per day) is also shown. n indicates the total number of participants in each group: Older group (n = 51), Control group (n = 56).

All patients received physical and occupational therapy while speech therapy was administered to 47.7% of the patients, with 45.1% in the older group and 50.0% in the control group (data were not shown). The median daily rehabilitation time at the rehabilitation hospital was 137.8 (interquartile range (IQR), 126.9-148.4) min, and the median length of hospitalization was 71 (IQR, 44-88) days (Table 1). No significant differences were found in daily rehabilitation duration or length of hospital stay between the groups. When examining specific components of the CR program, the older group engaged in significantly less aerobic exercise per day (median, 9.8 (IQR, 0.4-19.2) min) than the control group (median, 20.6 (IQR, 11.7-29.8) min, *P* < 0.001). No significant differences were observed in the daily duration of resistance training (26.5 (20.1-34.4) vs. 23.2 (18.6-29.1) min, *P* =0.094), balance training (3.2 (0.6-5.8) vs. 2.7 (0.5-8.2) min, *P* =0.353), ADL training (22.2 (16.5-30.8) vs. 18.5 (10.7-26.4) min, *P* =0.102), or conditioning training (31.7 (21.3-39.6) vs. 28.0 (19.9-40.9) min, *P* =0.953) per day between the two groups. Figure 2 provides a detailed breakdown of the percentages of CR implementation across the groups.

Changes in functional and clinical outcomes

Both groups exhibited significant improvements from transfer to discharge across multiple domains, including FIM, SPPB, gait speed, hand grip strength, knee extension strength, 6MD, MMSE, EQ-5D-5L, and MNA-SF scores (*P* < 0.001 for all; Table 2).

**Table 2:** Changes in ADL, physical function, exercise tolerance, cognitive function, and QoL after rehabilitation in all patients Results expression as median (IQR) ADL, activities of daily living; QoL, quality of life; FIM, functional independence measure; SPPB, short physical performance battery; 6MD, 6-min walking distance; MMSE, mini-mental state examination; EQ-5D-5L, EuroQol five-dimension five-level; MNA-SF, mini nutritional assessment short-form

	At transfer	At discharge	* P* value
FIM, points	70.0 (55.0 – 83.5)	118 (111 – 122)	<0.001
FIM-motor, points	45.0 (36.0 – 53.5)	84.0 (78.5 – 88.0)	<0.001
FIM-cognitive, points	25 (17 – 31)	35 (31 – 35)	<0.001
SPPB, points	7 (4 – 10)	12 (9 – 12)	<0.001
Gait speed, m/s	0.74 (0.57 – 0.98)	1.01 (0.81 – 1.25)	<0.001
Hand grip strength, kg	14.1 (10.8 – 20.6)	15.2 (11.7 – 21.6)	<0.001
Knee extension strength, %BW	26.8 (20.6 – 35.1)	34.3 (26.5 – 43.6)	<0.001
6MD, m	184.5 (91.0 – 283.8)	300.0 (200.0 – 403.8)	<0.001
MMSE, points	26 (24 – 28)	27.5 (25.0 – 29.0)	<0.001
EQ-5D-5L	0.638 (0.539 – 0.700)	0.750 (0.644 – 0.843)	<0.001
MNA-SF, points	6 (4 – 8)	11 (10 – 12)	<0.001

A two-way analysis of variance (ANOVA) revealed a significant interaction effect for only knee extension strength (older group, 26.4 (17.6-34.2) vs. 32.7 (25.8-41.8) %BW; control group, 27.0 (21.2-35.3) vs. 35.8 (27.4-45.9) %BW, P = 0.021). No significant interaction effects were observed for other measures, including FIM (total, motor, and cognitive subdomains), SPPB, gait speed, hand grip strength, 6MD, MMSE, EQ-5D-5L, and MNA-SF (Table 3).

**Table 3:** Comparison of the older and control groups *, *P* < 0.001, †, *P* < 0.01, ‡, *P* < 0.05; Comparison between groups at admission and discharge Results expression as median (IQR) FIM, functional independence measure; SPPB, short physical performance battery; 6MD, 6-min walking distance; MMSE, mini-mental state examination; EQ-5D-5L, EuroQol five-dimension five-level; MNA-SF, mini nutritional assessment short-form

		At transfer		At discharge		Main effect		Interaction	
						F-value	*P*-value	F-value	*P*-value
FIM, points	Older	62.0 (53.0 – 78.0)		115.0 (103.5 – 120.0) ^†^		1033.73	< 0.001	0.275	0.601
	Control	71.0 (58.8 – 85.5)		119.5 (114.8 – 124.0)					
FIM-motor, points	Older	41.0 (36.0 – 51.5)		84.0 (75.5 – 85.0) ^†^		1169.08	< 0.001	2.332	0.130
	Control	49.0 (36.8 – 56.0)		85.5 (83.0 – 89.0)					
FIM-cognitive, points	Older	22.0 (16.0 – 29.0) ^‡^		34.0 (30.5 – 35.0)		193.51	< 0.001	2.004	0.160
	Control	25.5 (18.8 – 31.3)		35.0 (32.8 – 35.0)					
SPPB, points	Older	6.0 (3.5 – 9.5)		10.0 (7.3 – 12.0) ^‡^		132.79	< 0.001	0.363	0.548
	Control	9.0 (4.8 – 11.0)		12.0 (10.5 – 12.0)					
Gait speed, m/s	Older	0.68 (0.49 – 0.85) *		0.92 (0.73 – 1.07) *		116.62	< 0.001	1.518	0.221
	Control	0.87 (0.63 – 1.09)		1.19 (0.91 – 1.35)					
Hand grip strength, kg	Older	12.7 (9.9 – 15.5) *		13.9 (10.8 – 16.6) *		20.62	< 0.001	1.225	0.271
	Control	16.2 (11.8 – 24.2)		18.6 (13.5 – 25.6)					
Knee extension strength, %BW	Older	26.4 (17.6 – 34.2)		32.7 (25.8 – 41.8)		87.61	< 0.001	4.209	0.043
	Control	27.0 (21.2 – 35.3)		35.8 (27.4 – 45.9)					
6MD, m	Older	120.0 (82.5 – 207.5) ^†^		250.0 (160.0 – 315.0) *		132.29	< 0.001	1.025	0.314
	Control	228.5 (129.3 – 314.3)		363.0 (272.0 – 433.5) *					
MMSE, points	Older	25.0 (24.0 – 28.0)		27.5 (25.0 – 29.0)		47.21	< 0.001	0.640	0.425
	Control	26.0 (23.8 – 29.0)		27.5 (25.0 – 29.0)					
EQ-5D-5L	Older	0.601 (0.504 – 0.688)		0.726 (0.646 – 0.814)		69.33	< 0.001	0.091	0.763
	Control	0.654 (0.564 – 0.708)		0.755 (0.644 – 0.843)					
MNA-SF, points	Older	6 (4 – 8)		10 (9 – 11)		406.97	< 0.001	0.341	0.561
	Control	6 (5 – 8)		11 (10 – 12)					

## Discussion

This study offers valuable insights into the effectiveness of CR for older patients in a convalescent rehabilitation hospital. Although the CR content for this population involves a shorter duration of aerobic exercise and requires individually tailored rehabilitation approaches, it contributes to improvements in ADL, physical function, and HR-QoL.

Most study participants were older patients, with a median age of 79 years, and predominantly female. Most patients had a relatively normal cardiac systolic function (median LVEF of 58.7%) and moderate severity (80.4% NYHA class 3). Although these patients are often independent in ADL before admission, they tend to present with frailty. These characteristics (Table 1) are typical of patients with CVD who require specialized rehabilitation following acute care [7]. The findings underscore the necessity of CR programs tailored to the diverse clinical needs of these patients.

In this study, CR continuation in a convalescent rehabilitation hospital led to significant improvements in ADL, physical function, cognitive function, exercise tolerance, and HR-QoL, regardless of age. These results are consistent with those of previous studies [7], showing that intensive CR during convalescent hospitalization significantly enhances clinical outcomes.

Compared with the control group, the older group had a higher proportion of women, more patients living alone, and a greater prevalence of frailty or prefrailty with poorer daily functioning, despite no differences in ADL before admission. Studies have suggested that women undergoing cardiac surgery tend to be older and in poorer preoperative condition, and experience higher rates of surgical complications and mortality, as well as reduced physical function after postoperative rehabilitation [19,20]. In addition, the J-Proof HF Registry revealed that those with HAD were older and had higher KCL scores before hospitalization [6]. This result aligns with our findings, where significantly lower physical function/performance and higher level of care required at discharge in the older group may have induced frailty before hospitalization or HAD in the acute care hospital. However, the inability to directly assess physical function prior to admission to an acute care hospital introduces a potential measurement bias that cannot be completely eliminated. The older group engaged significantly shorter durations of aerobic exercise as part of their CR. Given the prevalence of frailty among older patients, balance and mobility impairments must be addressed, as these factors can limit the effectiveness of standard endurance training following heart failure treatment [21]. Moreover, CR was reported to be less effective in older patients who underwent cardiac surgery [22]. Consequently, the CR content for this population involves a shorter duration of aerobic exercise and requires tailored rehabilitation approaches to meet individual needs.

Despite differences in CR contents, the two-way ANOVA revealed a significant interaction only for the degree of improvement in knee extension muscle strength, and no interactions were observed for improvements in ADL, physical function, cognitive function, HR-QoL, or nutritional status. The significant interaction effect for knee extension strength improvement could be attributed to age-related physiological differences. Compared to the younger group, the older group tended to experience a slower rate of muscle protein synthesis and inhibition of muscle hypertrophy in response to resistance training [23,24]. Previous studies have shown that intensive resistance training can lead to significant gains in knee extensor strength in frail nursing home residents with a mean age of 87 years [25]. Since there was no significant difference in the duration of resistance training in this study, increasing the frequency or intensity of resistance training may be a viable strategy to optimize muscle strength improvement in octogenarians undergoing CR. However, the older group in this study was predominantly female, and women may be more susceptible to age-related muscle weakness. Additionally, the presence of sarcopenia was not assessed, which may have influenced the outcomes. Therefore, additional muscle-strengthening strategies may be necessary for older women and patients with sarcopenia to improve rehabilitation outcomes. For other outcomes where no interactions were found, the older group still demonstrated notable improvements that exceeded the minimum clinically important difference previously reported [26,27], with a median increase of four points in the SPPB and 130 m in the 6MD. These findings indicate that positive changes occurred consistently across both groups from baseline to posttreatment, indicating that the older group achieved similar recovery levels to the control group. This highlights the utility of CR in rehabilitation hospitals, regardless of older patients. However, the median gait speed and 6MD at discharge in the older group were below the thresholds associated with poor prognosis in patients with heart failure [28,29]. This finding highlights the need for tailored medical and nursing care to ensure these patients can continue CR after discharge from the convalescent hospital. Tsuchihashi et al. reported that a lack of social support is linked to rehospitalization in patients with heart failure [30]. Therefore, an important role of rehabilitation hospitals is to optimize the discharge environment and coordinate preferred care services to support patients after discharge. Given that this study found that older women were more likely to live alone, they may have a greater need for structured post-discharge support than men.

Intensive CR in a convalescent hospital appears to be effective in all age groups, but its implementation should be tailored to the specific condition and needs of each patient.

Limitation

This study has several limitations. First, the sample size was relatively small owing to the limited capacity for long-term inpatient CR admissions. This small sample size may reduce the statistical power and limit the generalizability of the results. Second, the single rehabilitation hospital setting may restrict the applicability of the findings to other settings or populations, particularly those outside of similar healthcare environments. Third, the emphasis on needs-based rehabilitation programs may have introduced variability in interventions and heterogeneity, potentially limiting standardization and generalizability of findings to other groups or clinical settings. Fourth, both groups consisted of CR inpatients and the absence of a control or observation group without CR intervention makes it challenging to definitively attribute the observed improvements to rehabilitation hospitalization. Fifth, this study only evaluated short-term outcomes at discharge and did not include follow-up after discharge. Future research should evaluate long-term functional outcomes and readmission rates at one and three years to assess the lasting effects of CR. Finally, although a higher proportion of women were included in the older group, this study did not specifically analyze gender differences in the effectiveness of rehabilitation. Given the potential for gender differences in muscle strength gains, future studies should include gender-specific analyses to better understand differences in CR response.

## Conclusions

The findings of this study imply that CR for older patients in a convalescent rehabilitation hospital setting can improve ADL, physical function, and HR-QoL, despite the shorter duration of aerobic exercise. These results support the further development and implementation of inpatient CR programs in Japanese rehabilitation hospitals. Future research should examine long-term outcomes, assess the impact of post-discharge rehabilitation support, and evaluate strategies to further optimize the effectiveness of CR in older patients.
